# Bias Normalization for Sensors in Smart Devices

**DOI:** 10.3390/s25237291

**Published:** 2025-11-30

**Authors:** Wonjoon Son, Lynn Choi

**Affiliations:** School of Electrical Engineering, Korea University, Seoul 02841, Republic of Korea; swj8905@korea.ac.kr

**Keywords:** sensor bias, offset bias, scale bias, drift bias, bias normalization, indoor positioning

## Abstract

Modern electronic devices, such as smartphones and drones, integrate various sensors to enable diverse sensor-based applications. Yet, sensor measurements exhibit significant variations across different device models, even under the same environment. These variations arise from sensor biases, which occur in three different types: offset bias (additive constant errors), scale bias (multiplicative proportional errors), and drift bias (time-dependent or temperature-dependent errors). Among the biases, in this paper we specifically target offset bias, which has the greatest impact in typical smartphone usage scenarios. This generally leads to performance degradation in sensor-based applications across various device models and instances. To understand the characteristics of the offset bias, we categorize sensors into sensors with and without absolute reference values. Sensors with absolute references enable direct calibration using theoretical true values, while sensors with relative references require different approaches depending on how sensor applications process the data. For scalar-based applications that determine the current state by comparing a sensor measurement against a pre-defined reference, the offset biases can be removed by the existing procedures using reference devices. However, for sequence-based applications that determine the current state by analyzing relative changes in a sequence, the offset bias issue has not been addressed yet. We propose initial value removal and mean removal algorithms that statically and dynamically remove the offset biases from the sensor data sequences for these sequence-based applications. We evaluate our bias normalization algorithms for two different use cases in a geomagnetic-based indoor positioning system (IPS). First, we evaluate the impact of our bias normalization algorithms on the positioning performance of our LSTM-based IPS. Without bias normalization, although the reference device (Galaxy S23 Plus) showed an average positioning error of 0.6 m, the other three smartphone models (Galaxy S22 Plus, iPhone 15, and iPhone 16 Pro) exhibited much worse positioning performance, with errors of 2.48 m, 18.21 m, and 13.13 m. However, after applying our bias normalization, the average positioning errors of all models dropped below 0.68 m. Second, we also evaluate the impact of the bias normalization on detecting whether the position of a smartphone is in a pocket or in a hand-held state. For this, we analyze the sequence of light sensor measurements. We improved the detection accuracy from 42.3% to 97.6% with bias normalization across all device models without requiring individual threshold settings.

## 1. Introduction

A smartphone has now become a general-purpose computing device in modern society, evolving far beyond a simple communication device to play a critical role in daily life with numerous applications. A typical smartphone is equipped with various sensors, such as an accelerometer, a gyroscope, a magnetometer, a light sensor, a barometer, and sometimes even a LiDAR. It provides the foundation for a wide array of innovative services, such as location-based services (LBS) [1], augmented reality (AR) [2], healthcare [3], and smart homes [4]. The performance of these sensor-based applications depends heavily on the accuracy and consistency of the sensor data.

**Figure 1:**
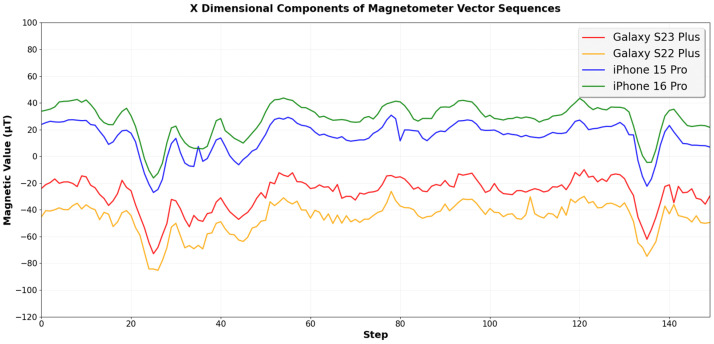
Comparison of magnetic field vector sequences in the x dimension collected from four different smartphone models along the same walking path, showing significant differences of up to 74 μT among the devices.

However, in a real-world environment, different smartphone models produce different sensor measurements even under the same conditions. Figure 1 shows the magnetic field vector sequences in the x dimension measured by four different smartphones (Galaxy S23 Plus (Samsung Electronics Co., Ltd., Suwon, Republic of Korea), Galaxy S22 Plus (Samsung Electronics Co., Ltd., Suwon, Republic of Korea), iPhone 15 Pro (Apple Inc., Cupertino, CA, USA), and iPhone 16 Pro (Apple Inc., Cupertino, CA, USA)) along the same 150-step walking path. The magnetic field measurements show a difference of up to 74 µT between the iPhone 16 Pro and the Galaxy S22 Plus.

These differences in magnetic field measurements can substantially degrade the performance of a geomagnetic vector-based indoor positioning system (IPS) [5,6]. This system operates in two phases: an offline phase and an online phase. In the offline phase, we first generate a magnetic field map by collecting magnetic field data from the indoor environment. In the online phase, our LSTM-based IPS uses consecutive magnetic field vector sequences as input to estimate the user’s location. Since the LSTM model is trained with the pre-collected magnetic field map, it achieves the best performance when the magnetic field values measured during the online phase closely match the training data from the magnetic field map. Since a Galaxy S23 Plus is used to collect the magnetic field map in the offline phase, the IPS shows the best positioning results when the same S23 Plus model is used in the online phase, with an average positioning error of only 0.60 m. However, in the cases of the Galaxy S22 Plus, iPhone 15 Pro, and iPhone 16 Pro, which show different magnetic field measurement values with differences of about 19.90 μT, 41,74 μT, and 56.47 μT, respectively, compared to the Galaxy S23 Plus, the average positioning errors increase to 2.48 m, 18.21 m, and 13.13 m, respectively.

**Figure 2:**
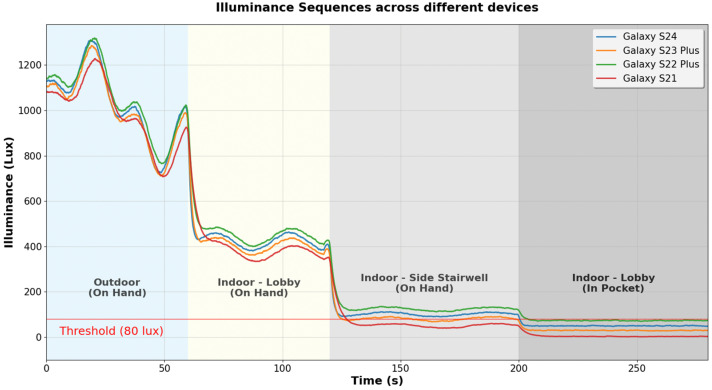
A comparison of light sensor measurements from four different smartphone models according to two different carrying positions (on-hand or in-pocket) and three different environments (outdoor, indoor lobby, or indoor side stairwell). The red line indicates the 80 Lux threshold set for in-pocket detection. Differences of up to 73 Lux in sensor measurements exist among the four smartphone models, potentially leading to misclassifications in carrying position detection.

The same problem exists for light sensors. Light sensors are often used to identify the user’s device carrying position (such as on-hand or in-pocket) or to identify the user’s environment (such as indoor or outdoor). Figure 2 shows the light sensor measurements collected under various environments with two different device carrying positions. Due to Apple’s API policies that restrict direct access to raw light sensor data from iOS [7], we show data collected from only Samsung Galaxy smartphone models. Light sensor measurements show differences across smartphone models, with up to a 73 Lux difference between the Galaxy S21 and the Galaxy S22 Plus, as shown in Figure 2. For example, if we use a threshold of 80 Lux to detect the on-hand position in an indoor environment with the Galaxy S22 Plus, the same condition would be incorrectly identified as in-pocket state with the Galaxy S21, since it would measure the same light at only 50 Lux.

These differences in sensor measurements among devices are called sensor biases. In Section 2, we categorize sensor biases into three different types and analyze the characteristics of each bias type and their impact on sensor measurements. Through this analysis, we argue that offset bias has the largest impact on sensor measurements compared to other bias types in a typical smartphone usage environment.

In Section 3, we discuss the existing bias removal procedures and algorithms. Since the removal methods vary depending on sensor and application characteristics, we first classify sensors into sensors with absolute references and those with relative references. Then, we classify applications into single-value-based applications and sequence-based applications. We discuss the existing removal methods and their limitations for each sensor and application type. Then, we define the coverage of our proposed offset bias normalization algorithm.

In Section 4, we present our offset bias normalization algorithm for sequence-based applications. The algorithm includes initial value removal and mean removal procedures that statically and dynamically remove offset bias from sensor data sequences.

In Section 5, we evaluate the performance of the proposed bias normalization algorithm in two different use cases in our geomagnetic vector-based IPS [5,6]. In this scenario, we address the positioning errors of the LSTM-based IPS caused by offset biases in magnetometer sensors with bias normalization, reducing the average positioning error from over 10 m to 0.65 m. We also apply the algorithm to smartphone carrying position detection. Due to offset biases in light sensors, each device previously required individual threshold settings for in-pocket or on-hand detection. However, with bias normalization, we could improve in-pocket detection accuracy from 42% to 97% by using only a single threshold across all devices.

## 2. Classification of Sensor Bias Types

Biases in sensor measurements are problematic and occur in all the sensors of a smartphone regardless of sensor type including even LiDAR, and RF (Radio Frequency) chipsets of wireless signals such as Wi-Fi or BLE. For example, in the case of the accelerometer and gyroscope, sensor biases can increase errors in step length estimation in PDR (Pedestrian Dead Reckoning) [8]. In addition, they affect the accuracy of attitude estimation in augmented reality (AR) applications [9]. In LiDAR-based SLAM [10], the biases in distance measurement cause mismatches between live scans and pre-collected point cloud maps, leading to unreliable position estimates. For RF chipsets measuring RSSI, the biases in signal strength measurement make fingerprint matching with pre-collected RF maps difficult, reducing positioning accuracy in RF-based IPS [11]. Therefore, minimizing biases in sensor measurements is essential for various sensor-based applications across diverse devices. To analyze sensor biases in detail, we need to understand various types of biases and their characteristics. Biases in sensor measurements can be categorized into three different types based on their characteristics [12]: offset bias, scale bias, and drift bias.

Offset bias is a phenomenon where a constant value is added to or subtracted from the sensor’s measurements. We express this bias with the following equation [12]:

$$x_{measured}\;=\;x_{true}\;+\;b_{offset}\;+\;\varepsilon_{noise},$$

where $x_{measured}$ is the measured value, $x_{true}$ is the true value, $b_{offset}$ is the offset bias, and $\varepsilon_{noise}$ is the noise. Offset bias is the most common and widespread type of bias, arising from factors such as manufacturing tolerances, differences in sensor installation, and variations in hardware component characteristics.

Scale bias is a multiplicative error where a sensor’s measurement is proportionally larger or smaller than the true value. We express this phenomenon with the following equation [12]:

$$x_{measured}\;=\;{(x}_{true}\;\times \;S_{factor})\;+\;\varepsilon_{noise},$$

where $S_{factor}$ represents the scale factor. A scale factor greater than 1 indicates that the sensor overestimates the magnitude of change, while a value less than 1 means it underestimates it. This type of bias often arises from differences in the gain of amplification circuits between devices. Unlike an offset, which uniformly shifts the entire signal, scale bias reflects differences in the sensor’s sensitivity.

Drift bias is a time-dependent and temperature-dependent error where a sensor’s offset gradually changes. We represent the bias with the following equation [12]:

$$x_{measured}\;=\;x_{true}+(d_{rate}\times t)\;+\;(T_{coeff}\times \;\Delta T)+\varepsilon_{noise},$$

where $d_{rate}$ is the drift rate and t is the elapsed time, $T_{coeff}$ is the temperature coefficient, and ΔT is the temperature change from the reference temperature. This type of bias is typically driven by slow-acting factors such as thermal variations within the device and the long-term aging of the sensor. Temperature changes affect the physical properties of sensor materials, causing variations in their electrical characteristics and measurement accuracy.

Table 1 summarizes bias specifications for sensor models commonly used in smartphones, referencing official datasheets [13,14,15,16] and experimental results from existing studies [17,18,19,20]. Since these values are measured under ideal laboratory conditions, in an actual environment, a sensor can exhibit 5–20 times larger bias due to interference from other components, manufacturing tolerances, and lack of calibration [17,18,19,20].

By comparing the relative magnitudes of offset bias, scale bias, and drift bias in this table, we can determine which bias type has the greatest impact on sensor measurements. Offset bias has an immediate and direct impact on sensors. For example, the accelerometer’s offset bias of ±0.02 m/s^2^ and the gyroscope’s offset bias of ±0.0035 rad/s adds constant error to all measurements. This causes non-zero outputs even in stationary states, potentially creating problems for application performance. For instance, even when a smartphone is completely stationary, the gyroscope’s offset bias causes the system to perceive that the device is rotating.

In contrast, drift bias and scale bias have a relatively smaller impact on sensor measurements in typical smartphone usage environments. Drift bias shows noticeable effects only under extreme conditions, such as when temperature differences exceed 20 degrees or when a smartphone operates continuously for over 1–2 years. For example, in the gyroscope, temperature-induced drift generates only ±0.00052 rad/s error for a 10 °C temperature change, which is 6.7 times smaller than the continuously acting offset bias (±0.0035 rad/s).

Scale bias has a smaller impact than offset bias in typical smartphone usage environments, such as walking, sitting, and running. For example, the accelerometer typically shows peak values of 1–3 m/s^2^ unless riding a roller coaster or experiencing vehicle collisions, resulting in scale bias errors of 0.005–0.015 m/s^2^, which are two to six times smaller than offset bias (0.02 m/s^2^).

In conclusion, offset bias affects sensor measurements more than other bias types in a typical smartphone usage environment. In this paper, we focus on offset bias, which has the greatest impact on sensor measurements and their applications.

## 3. Classification of Sensors and Applications in Terms of Biases

### 3.1. Sensors with Absolute Reference and Relative Reference

Offset bias is the difference between the measured value and the true value. Therefore, to calculate offset bias, we need to know the true value of the sensor. As an example, a gyroscope in a stationary state must measure exactly 0 rad/s according to physical law. If 0.1 rad/s is measured, the offset bias should be 0.1 rad/s. In contrast, a magnetometer is different. For example, if 50 μT is measured at the current location, we cannot directly calculate the offset bias unless we have an ideal magnetometer sensor showing the true value of the magnetic field. Therefore, removing the offset bias depends on whether we can determine the true value. Sensors with known true values enable direct calibration, while sensors without known true values require indirect methods using relative changes or measuring offsets with reference devices. Therefore, we classify sensors into sensors with absolute reference and relative reference sensors and introduce existing studies for removing offset bias for each sensor type.

Sensors with absolute references are those that have clear theoretical true values based on physical laws. The key characteristic of these sensors is that they should always measure the same value under specific conditions, regardless of external environment changes.

The gyroscope is a representative sensor with an absolute reference. The angular velocity for all axes (x, y, z) must be exactly 0 rad/s when the device is in a completely stationary state with no rotational movement, according to physical laws. Similarly, for a linear accelerometer, which measures only acceleration velocity without the gravity component, the acceleration on all axes should be 0 m/s^2^ when the device is perfectly still.

The existence of this absolute reference value makes bias measurement straightforward. If the gyroscope or accelerometer outputs non-zero values in a stationary state, the difference can be directly estimated as the sensor’s offset bias. This allows accurate bias measurement on individual devices without external references.

Unlike the accelerometer and gyroscope, sensors such as a magnetometer, a barometer, and a light sensor present a different challenge for offset bias measurement. While these sensors theoretically have absolute reference values—zero magnetic field in a magnetically shielded room, zero pressure in a vacuum, and zero Lux in complete darkness—it is not easy to create such conditions. For example, creating a zero magnetic field environment requires specialized shielding facilities. Given these practical limitations, it is not easy to measure the absolute offset bias of these sensors. However, we can still measure the relative offset bias between devices. The relative offset bias is measured by comparing measurements of a device with those of a reference device under the same condition.

Applications using this type of sensor need to minimize the relative offset biases among devices to ensure consistent performance. In the following section, we further classify these applications into two distinct categories and discuss bias normalization strategies for each category.

### 3.2. Classification of Applications with Relative Reference Sensors

We classify these applications into two types: scalar-based and sequence-based. Scalar-based applications determine the current state by comparing the value of a sensor measurement against a pre-defined reference. For example, the in-pocket status of a smartphone can be detected using a light sensor [21] if the measurement falls below a pre-defined threshold. KNN-based indoor positioning systems [22,23] estimate the current location by comparing the RSSI values from radio signals such as Wi-Fi or BLE to those of reference points in the pre-collected map.

To address the offset bias in these scalar-based applications, existing studies [24,25] employed a reference device. For example, in a geomagnetic field-based IPS, the smartphone model used to collect the magnetic field map during the offline phase serves as the reference device [24]. If a user uses a different smartphone model during the online phase, its offset bias relative to the reference device will create mismatches, resulting in reduced positioning performance. To avoid such mismatches, we measured the offset bias of each smartphone model using the reference device in advance during the offline phase, which we call static bias removal. Then, during the online phase, this offset bias is subtracted from the device’s sensor measurements to reduce the mismatches.

In contrast, sequence-based applications analyze the temporal patterns or relative changes in a series of consecutive sensor measurements to determine the current state or location. For example, to detect a floor change, we commonly use a barometer and analyze a sequence of measurements to estimate how many floors the user has moved. Likewise, an RNN/LSTM-based geomagnetic IPS [5,6] estimates the user’s current location by analyzing the sequence of magnetic field vectors.

Note that offset bias only shifts the entire sequence uniformly up or down. Therefore, even though each sensor measurement creates a difference due to the offset bias, the entire sequence generated by each device shows almost the same waveform under the same environment. Therefore, by analyzing the relative changes between measurements, we can eliminate the impact of offset bias on application performance.

We propose bias normalization algorithms that can be applied to sequence-based applications, overcoming the limitation of existing approaches that simply use relative changes. Our algorithms statically and dynamically remove offset bias from entire sequences, allowing real-time bias normalization across different devices and maintaining consistent application performance without prior calibration or external data.

## 4. Offset Bias Normalization Algorithms for Sequence-Based Applications

We propose initial value removal and mean removal algorithms to effectively remove offset bias for sequence-based applications. The initial value removal algorithm uses the first measurement in a sequence as a baseline and subtracts it from all subsequent measurements. This can be expressed as:

$$x_{normalized}(t)\;=\;x_{measured}(t)-\;x_{measured}(1),\;where\;t\;=\;1,\;2,\;3,\;...,\;N$$

where $x_{measured}(t)$ is the original measured value at time t, $x_{normalized}(t)$ is the normalized value, $x_{measured}(1)$ is the first measured value of the sequence, and N is the total length of the sequence. This method is simple to implement and appropriate for real-time processing, requiring O(1) memory (only the initial value) and O(1) time complexity (one subtraction per measurement).

**Figure 3:**
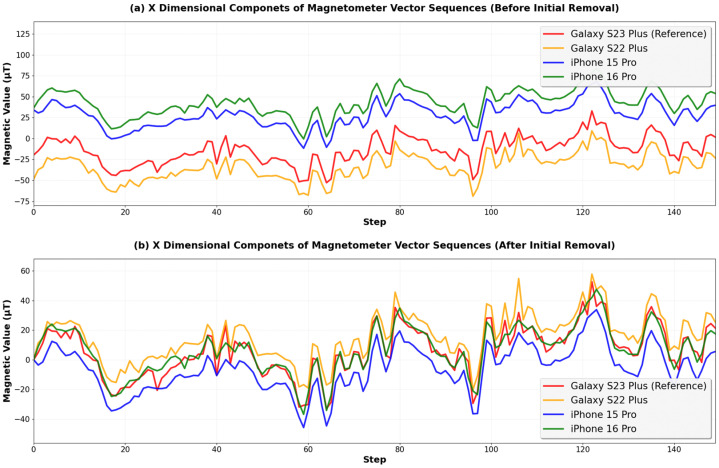
Comparison of magnetic field vector sequences in the x dimension before and after applying the initial value removal algorithm. (**a**) Original magnetic field vector sequences collected from four different smartphone models along the same 150-step walking path, showing offset biases among the devices. (**b**) Magnetic field vector sequences after applying the initial value removal algorithm, demonstrating reduced offset biases among the devices.

Figure 3 shows the results of applying the initial value removal algorithm to magnetic field vector sequences in the x dimension. Figure 3a shows the sequences from four different smartphone models along the same 150-step walking path. Before applying initial value removal, the offset biases relative to the reference device were 56.47 μT, 41.74 μT, and 19.90 μT for the iPhone 16 Pro, iPhone 15 Pro, and Galaxy S22 Plus, respectively. After the initial value removal, the biases decreased to 3.62 μT, 12.10 μT, and 9.08 μT as shown in Figure 3b.

However, the initial value removal relies on a single measurement. If the initial value contains noise or measurement error, the bias normalization performance degrades for the entire sequence. For example, in Figure 3a, the initial value of the iPhone 15 Pro differs from the reference device by 53.8 μT. This exceeds the actual offset bias differences with the reference device (41.74 μT) by 12.06 μT. This is due to slight orientation changes during the initial measurement since a user can change his or her smartphone orientation anytime during the online phase. However, the accurate offset bias of a smartphone model against a reference device should be measured with the exact same orientation in the three-dimensional spaces. This difference in the initial value affects all subsequent measurements in the sequence.

To address this limitation, we propose the mean removal algorithm. This algorithm calculates the mean of an initial sequence and subtracts it from all measurements. This is expressed as follows:

$$x_{normalized}(t)\;=\;x_{measured}(t)\;-\;\mu (N),\;\;\;where\;\mu (N)\;=\;\frac{1}{N}\;\sum_{i\;=\;1}^{N}x_{measured}(i)$$

where μ(N) represents the mean of an N-length sequence. Although this calculation has O(N) time complexity for a complete sequence, we can implement O(1) time complexity using the following equation:

$$\mu (N)=\;((N-1)\times \mu (N-1)\;+\;x_{measured}(N))\;/\;N$$

where μ(N) is the updated mean, μ(N−1) is the previous mean, $x_{measured}(N)$ is the measurement, and *N* is the current sequence length. The pseudo-code of the mean removal algorithm is shown in Algorithm 1.
**Algorithm 1**: The mean removal algorithm   **Input: **         Sensor measurement stream $x_{measured}(t)$   **Output:**        $x_{normalized}(t),\;\;t\;=\;1,\;2,\;3,\;\ldots$ 1.  mean = 0 2.  count = 0 3.  while **a** new measurement $x_{measured}(t)$ arrives: 4.      count = count + 1 5.      mean = mean + ($x_{measured}(t)$ − mean)/count 6.      $x_{normalized}(t)$ = $x_{measured}(t)$ − mean

**Figure 4:**
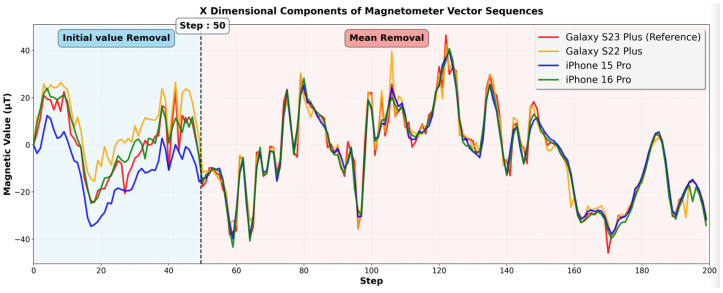
Magnetic field vector sequences during 200 steps in the x dimension after applying the hybrid approach. The initial value removal was applied for the first 50 steps, and the mean removal was applied after the 51st step. The offset biases are significantly reduced compared to the initial value removal results, demonstrating their effectiveness in all tested smartphone models.

Figure 4 shows the results of applying the mean removal algorithm to the sequences in Figure 3a. For the first 50 steps, the initial value removal was applied, and then from the 51st step, the mean removal was applied. After applying the mean removal, the offset biases were reduced to 2.81 μT, 3.78 μT, and 3.59 μT for the iPhone 16 Pro, iPhone 15 Pro, and Galaxy S22 Plus, respectively. Table 2 summarizes the results of applying the initial value removal and mean removal. This demonstrates that the mean removal algorithm is more robust against noise, device orientation changes, and external environment variations.

However, the mean removal algorithm requires a sufficient sequence length to effectively remove offset bias. With short sequences, a few noisy measurements can significantly distort the sequence mean, causing unstable normalization. For example, in a 200-length sequence, one or two noisy measurements, which can be caused by a metal object passing nearby or abrupt device shaking, have minimal impact on the mean, maintaining stable normalization. In contrast, in a 5-length sequence, the noisy measurements significantly change the mean, producing inconsistent bias normalization. However, the longer the sequence, the slower it is to apply the mean removal algorithm in practice. To address this issue, we use a hybrid approach, as illustrated in Figure 4. We apply the initial value removal initially when the sequence length is short and then transition to the mean removal as the sequence length increases.

**Figure 5:**
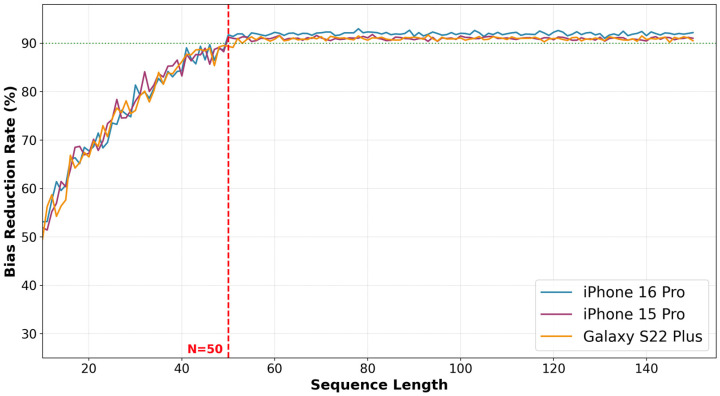
Bias reduction rates as we increase the sequence length for the mean removal algorithm. We use the Galaxy S23 Plus as the reference device and collect the results for three smartphone models (iPhone 16 Pro, iPhone 15 Pro, and Galaxy S22 Plus). The bias reduction rates steadily increase from 50% to 90% as we increase the sequence length from 10 to 50. Beyond a length of 50, the rates saturate and converge to approximately 91 to 92% across all devices. The green dashed line represents the 90% bias reduction threshold, included as a visual reference for the saturation region.

To determine the appropriate sequence length for the transition, we evaluated the impact of the sequence length on bias normalization by varying the length from 10 to 150. We inserted five random outlier values into each sequence and measured the bias reduction rate for each sequence length. The bias reduction rate shows how much of the offset bias has been removed after applying the mean removal. Figure 5 shows the bias reduction rates for three smartphone models (iPhone 16 Pro, iPhone 15 Pro, and Galaxy S22 Plus) in terms of the sequence length when we use the Galaxy S23 Plus as the reference device. As the sequence length increases from 10 to 50, the bias reduction rates steadily rise from 50% to 90%. When the sequence length exceeds 50, the bias reduction rates gradually saturate and converge. For the iPhone 16 Pro, iPhone 15 Pro, and Galaxy S22 Plus, the rates converge to 91.9%, 91.1%, and 90.6%, respectively. Therefore, we select 50 as the transition threshold, where the bias reduction rate exceeds 90%. The first 50-step length is long enough to provide robustness against noise. This hybrid approach effectively combines the initial removal algorithm with the mean removal algorithm, providing both robustness against noise and instant bias removal from the start.

## 5. Evaluation

### 5.1. Geomagnetic Field-Based IPS

**Figure 6:**
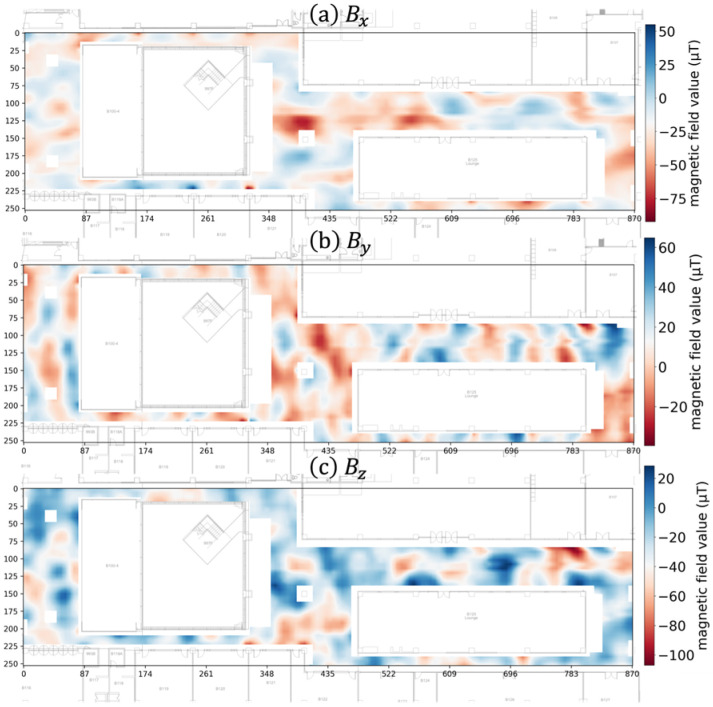
Three-dimensional magnetic field map of the Hana Square testbed. The map shows the collected magnetic field vector components interpolated at 10 cm grid intervals: (**a**) x dimension ($B_{x}$), (**b**) y dimension ($B_{y}$), and (**c**) z dimension ($B_{z}$).

We applied our bias normalization algorithms to our geomagnetic vector-based IPS [5,6]. We built the IPS in the Hana Square underground facility at Korea University Science Campus, which has an area of 2476 m^2^ (26 m × 95 m). We collected three-dimensional magnetic field vectors at 60 cm intervals using the Galaxy S23 Plus as the reference device. Then, we generated a magnetic field map, as shown in Figure 6, by interpolating the collected magnetic field data at 10 cm grid intervals.

**Figure 7:**
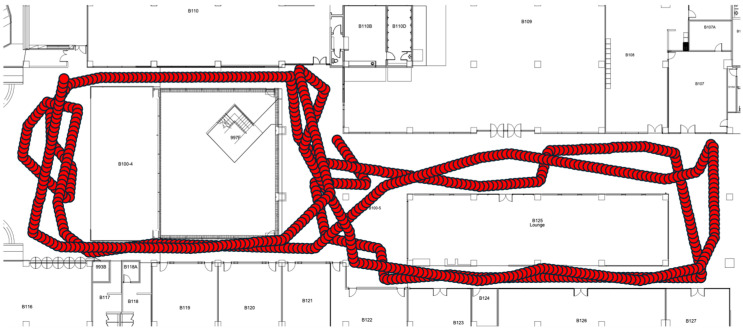
An example of a random path. The path consists of 100 consecutive (*x*,*y*) coordinates with random direction changes, simulating realistic human walking patterns within the testbed.

For positioning, i.e., mapping, we employed an LSTM model [6] by using geomagnetic vector sequences as input. Unlike a basic RNN model, an LSTM model can address long-term dependency problems [26], enabling higher positioning performance with longer vector sequences. The LSTM model requires training data consisting of magnetic field vector sequences paired with their corresponding position coordinates. To generate this data, we created random paths using a modified version of the random waypoint model [27]. Figure 7 shows one of the generated paths. We generated 400,000 paths of 100 steps each. For each coordinate, we extracted the corresponding three-dimensional magnetic field vector from the pre-collected magnetic field map. We used supervised learning with a 100 magnetic field vector sequence as an input and the corresponding final coordinate as the output. From the 400,000 datasets, we used 60% for training, 20% for validation, and 20% for testing.

We trained two LSTM models with two bias normalization algorithms. The first model used the training data processed with the initial value removal, and the second model used the training data processed with the mean removal. The reason for training two separate models is as follows.

During the real-time positioning phase, we use the hybrid approach to remove biases depending on the length of the magnetic field vector sequences. For the LSTM model to accurately estimate a position using the normalized sequences, both the training and test data must be processed using the same normalization method. If a sequence normalized with the initial value removal is used as input to a model trained with mean removal-processed data, the positioning error would increase significantly. This is because, even for magnetic field sequences measured along the same path, the actual magnetic field vector values themselves can vary depending on the normalization method. For example, the initial value removal normalizes based on the first value of the sequence, so the first value of the sequence is always 0, whereas the mean removal normalizes based on the average of the entire sequence, so the first value may be a non-zero value. Even if the sequence patterns are identical, if the magnetic field vector values themselves are different, the LSTM model will produce completely different position estimates. Therefore, we separately trained two LSTM models optimized for each normalization method. After training, during real-time positioning, we use the first model for sequences up to a length of 50, then switch to the second model for sequences of length greater than 50. Both LSTM models consist of three hidden layers with 300 hidden nodes each. Table 3 summarizes the detailed hyperparameters used for the models.

After training, the LSTM model trained with initial value removal achieved an average positioning error of 0.92 m, while the model trained with mean removal achieved an average positioning error of 0.49 m on the test set. However, note that this is for an offline test on the test dataset. In real-time tests with non-reference devices, positioning errors would increase due to offset biases and environmental noise.

We performed real-time positioning tests on six different test paths using the Galaxy S22 Plus, iPhone 16 Pro, and iPhone 15 Pro, including the reference device the Galaxy S23 Plus. Each test path consisted of 200 steps and included various direction changes and movement patterns. The ground truth for each test path was generated by manually recording the coordinates of each step along the actual walking path. For each path, we independently tested four smartphone models, with the user holding each device in portrait mode in their hand and walking at normal walking speed. The positioning error at each step was calculated as the Euclidean distance between the coordinate predicted by the LSTM model and the ground truth coordinate.

**Figure 8:**
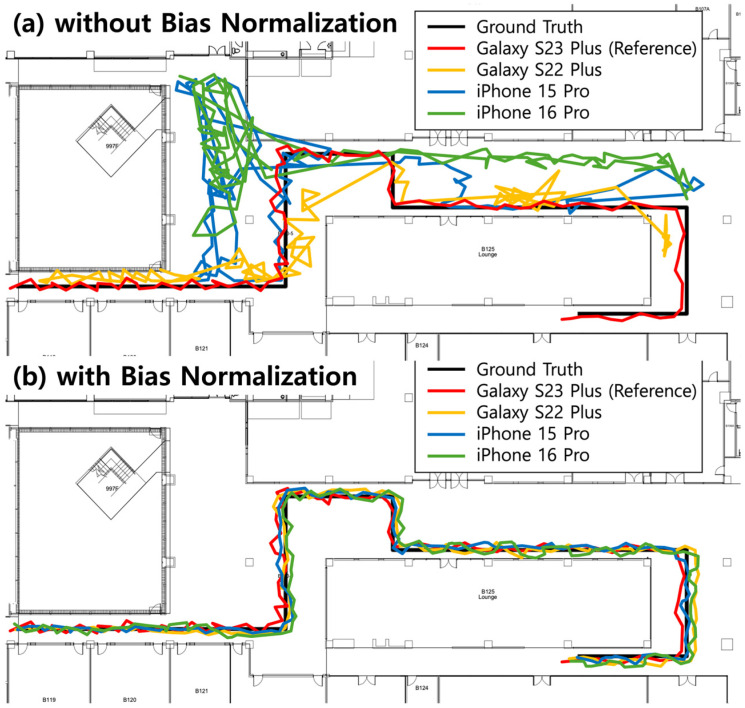
Real-time positioning results comparison for one test path. The black line represents the ground truth path, while colored lines show the positioning results predicted by the LSTM model for various smartphone models. (**a**) Results without bias normalization show degraded positioning performance for other smartphone models except the reference device. (**b**) Results with bias normalization show that all models produce improved positioning results with sub-meter accuracy.

Figure 8 shows the real-time positioning test results for one test path. The black line represents the actual path, and the colored lines show the positioning results with various smartphone models. Figure 8a shows positioning results without bias normalization, where we used an LSTM model trained with original magnetic field vector sequences collected from the reference device (Galaxy S23 Plus). The reference device (red line) achieved an average positioning error of 0.60 m. Galaxy S22 Plus (orange line), which has a relatively smaller offset bias compared to other models (19.9 μT), achieved an average positioning error of 2.48 m without normalization. In contrast, iPhone 15 Pro (blue line) and iPhone 16 Pro (green line) showed severe degradation with average positioning errors of 18.21 m and 13.13 m, respectively, due to their larger offset biases (41.74 μT and 56.47 μT).

Figure 8b shows the results with bias normalization. During the real-time positioning, we applied the initial value removal for sequences up to a length of 50 using the first LSTM model, then transitioned to the mean removal for sequences of length greater than 50 using the second LSTM model. All device models achieved sub-meter accuracy with average positioning errors of 0.64 m (Galaxy S23 Plus), 0.62 m (Galaxy S22 Plus), 0.68 m (iPhone 16 Pro), and 0.66 m (iPhone 15 Pro). Although the positioning error of the reference device has slightly increased from 0.60 m to 0.64 m, our bias normalization methods achieved consistent performance across all devices. The minor degradation in the positioning performance of the reference device is marginal and within the expected range of variation, which can naturally occur due to changes in walking patterns or sensor noise. Table 4 presents the detailed results for all six test paths.

### 5.2. Detection of Device Position

Determining whether a smartphone is in a pocket or held in hand is important for pedestrian dead reckoning (PDR) in indoor positioning. PDR estimates step length and direction of movement using the accelerometer and gyroscope sensor data [28]. The same walking motion produces different accelerometer patterns depending on smartphone position (in-pocket or hand-held). Therefore, detecting the carrying position of a smartphone is important for accurate step length and orientation estimation.

The existing study [21] uses single-value-based methods that compare an individual light sensor measurement against a pre-defined threshold to distinguish between in-pocket and hand-held states. However, this approach may fail when the pre-defined threshold cannot be applied to other devices due to offset biases.

**Figure 9:**
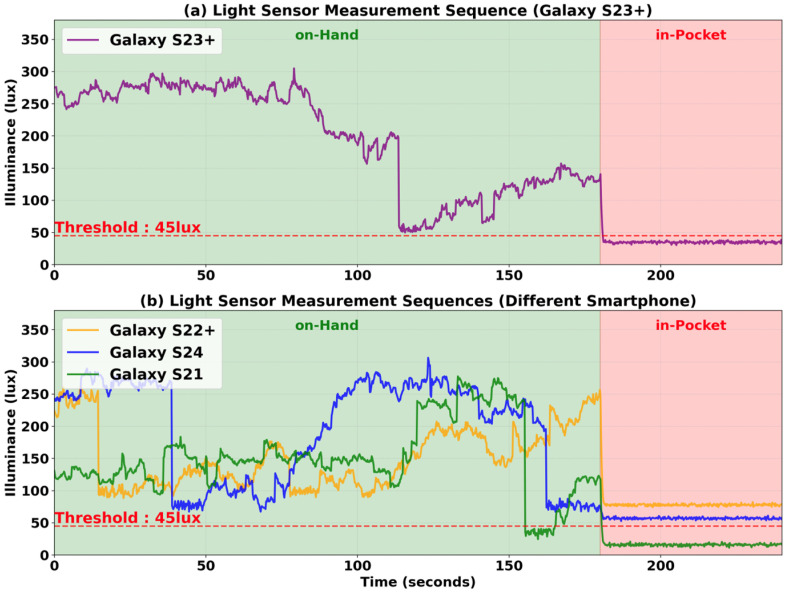
Light sensor measurements for on-hand and in-pocket states. (**a**) Galaxy S23 Plus: While held in hand, light sensor measurements ranged from approximately 55–310 Lux, and while in a pocket, the measurements were around 38 Lux. (**b**) Different smartphone models: Applying the same 45 Lux threshold leads to detection errors due to offset biases. For the Galaxy S22 Plus and Galaxy S24, the light sensor measurements exceeded the threshold, failing to detect the in-pocket state, while for the Galaxy S21, when held in hand, the light sensor measurements dropped below the threshold, leading to incorrect detection of the in-pocket state.

Figure 9a shows the light sensor measurements from the Galaxy S23 Plus while walking around in an indoor environment. The user held the smartphone in hand for 180 s and then placed it in a pocket. When held in hand, the light sensor measurements are approximately in the 55 to 310 Lux range. However, when placed in a pocket, the light sensor measurement was 38 Lux. To distinguish the smartphone position, we used a 45 Lux threshold.

However, as shown in Figure 9b, the in-pocket detection accuracy decreases due to offset biases. For the Galaxy S22 Plus and Galaxy S24, despite being placed in a pocket, the light sensor measurements were 78 Lux and 57 Lux, respectively, exceeding the 45 Lux threshold, failing to detect the in-pocket state. In contrast, for the Galaxy S21, the light sensor measurements were 16 Lux when placed in a pocket, correctly detecting the in-pocket state. However, between 155 and 164 s, the light sensor measurements decreased to 32 Lux, incorrectly indicating an in-pocket state even though the device was held in hand. Therefore, this single-threshold approach obviously does not work, requiring either measuring offset bias for each smartphone model individually or using different thresholds for each device model.

To address this, we analyze light sensor measurement sequences over time and apply mean removal to normalize device-specific offset biases. This allows us to use a single threshold across all devices.

However, this approach cannot detect the states immediately. It needs sufficient measurements to establish a baseline for normalization. If a user places a smartphone in a pocket immediately after starting the application, detection fails due to insufficient data. Despite this limitation, the sequence-based approach is robust to environmental changes and noise, allowing it to accurately distinguish whether the carrying position has moved from hand-held to in-pocket or whether the light sensor measurement has decreased due to a shadow. This robustness results from mean removal by eliminating device-specific offset biases, enabling different device models to share a single threshold.

**Figure 10:**
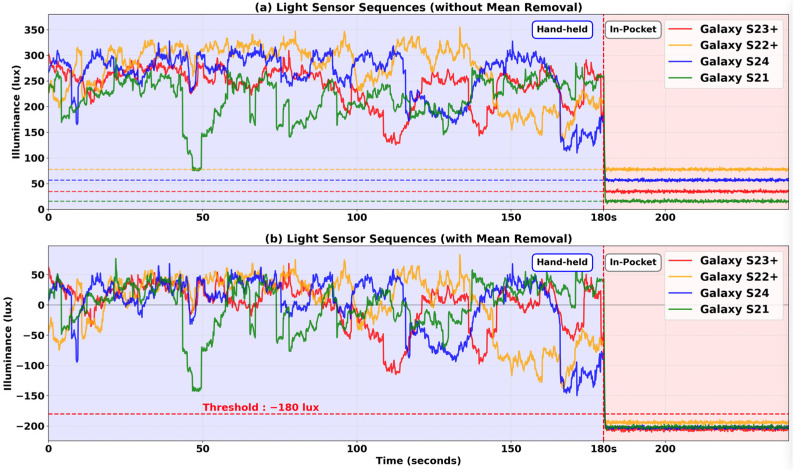
Comparison of in-pocket detection performance with different smartphone models. (**a**) Light sensor measurements without bias normalization show significant differences in values in the pocket across devices. (**b**) Mean removal bias normalization enables four smartphones to achieve consistent in-pocket detection, with all light sensor measurements converging below the −180 Lux threshold.

To evaluate our bias normalization algorithm for in-pocket or hand-held state detection, we collected light sensor measurements in the testbed while moving under bright environments (indoor lobby with natural light) and under darker environments (indoor corridors, stairwells). In each test, we held the smartphone in hand in portrait mode for 180 s while walking and then placed it in the front pants pocket. Figure 10a shows the light sensor measurements without bias normalization. When each device is placed in the pocket, different illuminance values are measured due to light sensor offset biases. In the pocket, the Galaxy S23 Plus, Galaxy S22 Plus, Galaxy S24, and Galaxy S21 measured approximately 35 Lux, 78 Lux, 57 Lux, and 16 Lux, respectively. Each smartphone model would require a separate threshold setting for accurate in-pocket detection.

In contrast, Figure 10b shows the results after applying mean removal bias normalization. The mean values of light sensor measurements were 261 Lux, 296 Lux, 278 Lux, and 238 Lux for the Galaxy S23 Plus, Galaxy S22 Plus, Galaxy S24, and Galaxy S21, respectively. After subtracting these means from the measurements, the normalized values of all the models in the hand-held state ranged from −182 Lux to +70 Lux, while they ranged from −202 Lux to −178 Lux in the in-pocket state. A value of −180 Lux was selected as the threshold, since it can clearly distinguish the hand-held state from the in-pocket state. As a result, Figure 10b demonstrates that the mean removal effectively removes the offset biases from various models, enabling robust smartphone position detection with a single threshold.

Table 5 compares the detection performance of in-pocket and hand-held states with and without applying mean removal. Without mean removal, we used a 45 Lux threshold using Galaxy S23 Plus as a reference device and tested this threshold on three other smartphone models. The reference device (Galaxy S23 Plus) achieved 99.89% accuracy. However, the other models achieved 33.92% (Galaxy S22 Plus), 39.46% (Galaxy S24), and 53.48% (Galaxy S21) accuracy. With mean removal, the four smartphone models used −180 Lux as a single threshold. The detection accuracy was improved to over 97% for all the models, showing that the mean removal effectively eliminates offset biases.

## 6. Conclusions

In this paper, we propose bias normalization methods that can address sensor measurement variations across different device models. First, we classify sensor biases into three different types: offset bias, scale bias, and drift bias. By analyzing each type of bias, we conclude that the offset bias has the largest impact in applications. Second, to target the offset bias, we further classify sensors with absolute reference and relative reference and suggest different bias normalization approaches for the two sensor types. We then categorize applications into scalar-based and sequence-based applications. Third, we propose initial value removal and mean removal algorithms for sequence-based applications. These methods normalize entire sequences rather than individual differences, maintaining pattern integrity while removing offset biases. Initial value removal provides immediate normalization for rapid convergence, while mean removal achieves superior accuracy after accumulating sufficient data. Our hybrid approach combines both advantages.

We evaluated the performance of our proposed methods on two different use cases in a geomagnetic vector-based IPS. First, we evaluate the performance of our proposed bias normalization algorithms on the positioning performance of LSTM models. Although the reference device (Galaxy S23 Plus) achieved the average positioning error of 0.6 m, other models showed significantly larger positioning errors ranging from 2.48 m (Galaxy S22 Plus) to 18.21 m (iPhone 15 Pro) due to the offset biases. However, after applying our normalization algorithms, we could reduce the positioning errors of all models below 0.68 m, effectively eliminating device-dependent performance variations. Second, we also evaluated the performance impact of our bias removal algorithms in detecting smartphone position. To detect the in-pocket or hand-held state using light sensor measurement sequences, we improved the average detection accuracy from 42.3% to 97.6% by applying the bias normalization algorithms for all the smartphone models using a single threshold, removing the need for device-specific calibration.

These results demonstrate that our proposed algorithms effectively remove the offset biases in sequence-based applications. The same algorithms can be applied to other sequence-based applications regardless of applications, including RF-based indoor positioning and floor movement detection with a barometer sensor. By ensuring consistent measurements across devices without individual calibration, our algorithms enable sensor applications to work reliably across different device models.

## Figures and Tables

**Table 1:** Smartphone sensors’ bias specifications [13,14,15,16,17,18,19,20].

Sensor	Model	Offset Bias	Scale Bias	Drift Bias
Accelerometer	ST LSM6DS3TR-C (STMicroelectronics N.V., Geneva, Switzerland)	±0.03 m/s^2^	±0.5%	±0.00049 m/s^2^/°C ±0.0098 m/s^2^/year
Gyroscope	ST LSM6DS3TR-C (STMicroelectronics N.V., Geneva, Switzerland)	±0.0035 rad/s	±0.5%	±0.000052 rad/s/°C ±0.00087 rad/year
Magnetometer	ST LIS3MDL (STMicroelectronics N.V., Geneva, Switzerland)	±5 µT	±1%	±0.002 µT/°C ±0.01 µT/year
Barometer	Bosch BMP280 (Robert Bosch GmbH, Gerlingen, Germany)	±0.5 hPa	±0.5%	±0.0015 hPa/°C ±0.01 hPa/year
Light Sensor	AMS TSL2591 (ams-OSRAM AG, Premstätten, Austria)	±5 Lux	±10%	±0.005 Lux/°C ±0.2 Lux/year
Wi-Fi (RSSI)	Qualcomm QCA6174A (Qualcomm Technologies, Inc., San Diego, CA, USA)	±5 dBm	±0.1%	±0.02 dBm/°C

**Table 2:** Comparison of offset bias reduction using initial value removal and mean removal algorithms.

vs. Galaxy S23 Plus (Reference)	Without Bias Normalization	With Initial Value Removal	With Mean Removal
iPhone 16 Pro	56.47 μT	3.62 μT	2.81 μT
iPhone 15 Pro	41.74 μT	12.1 μT	3.78 μT
Galaxy S22 Plus	19.9 μT	9.08 μT	3.59 μT

**Table 3:** Hyperparameters of the LSTM models.

Hyperparameter	Value
Hidden nodes, Hidden layer	600, 3
Batch size	300
Learning rate	0.0005
Loss function	Mean squared error
Optimizer	Adam
Activation function	tanh/ReLU
Dropout rate	0.2
Early stopping patience	50
Data split	60% (Train):20% (Validation):20% (Test)
Training epochs	500
Sequence length	100
Input dimension	3 (three-dimensional magnetic field vector)
Output dimension	2 (x, y coordinates)

**Table 4:** Real-time positioning errors for all test paths with and without bias normalization.

	Without Bias Normalization							With Bias Normalization						
	Test #1	Test #2	Test #3	Test #4	Test #5	Test #6	Avg.	Test #1	Test #2	Test #3	Test #4	Test #5	Test #6	Avg.
Galaxy S23 Plus (Reference)	0.58 m	0.63 m	0.58 m	0.65 m	0.51 m	0.67 m	0.60 m	0.64 m	0.72 m	0.57 m	0.71 m	0.61 m	0.59 m	0.64 m
Galaxy S22 Plus	1.26 m	1.91 m	4.31 m	2.18 m	2.17 m	3.05 m	2.48 m	0.55 m	0.65 m	0.45 m	0.76 m	0.68 m	0.61 m	0.62 m
iPhone 16 Pro	15.4 m	11.90 m	6.38 m	13.64 m	21.02 m	10.45 m	13.13 m	0.70 m	0.61 m	0.64 m	0.82 m	0.59 m	0.71 m	0.68 m
iPhone 15 Pro	30.51 m	15.81 m	14.32 m	11.52 m	18.19 m	18.88 m	18.21 m	0.65 m	0.71 m	0.62 m	0.79 m	0.68 m	0.51 m	0.66 m

**Table 5:** Detection of a smartphone position for four different smartphone models with and without the mean removal algorithm.

	Without Mean Removal						With Mean Removal					
	Test #1	Test #2	Test #3	Test #4	Test #5	Avg.	Test #1	Test #2	Test #3	Test #4	Test #5	Avg.
**Galaxy** **S23 Plus**	100%	99.82%	99.71%	100%	99.91%	99.89%	100%	100%	99.8%	100%	99.9%	99.94%
**Galaxy S22 Plus**	64.21%	21.64%	16.75%	24.88%	42.11%	33.92%	99.82%	98.76%	94.43%	95.74%	97.19%	97.19%
**Galaxy S24**	66.58%	29.81%	18.51%	27.81%	54.52%	39.46%	98.94%	99.01%	97.34%	97.11%	98.04%	98.09%
**Galaxy S21**	60.2%	26.79%	47.2%	61.71%	71.5%	53.48%	97.6%	98.74%	96.55%	97.42%	98.02%	97.67%

## Data Availability

The original contributions presented in this study are included in the article. Further inquiries can be directed to the corresponding author.

## References

[1] Raper J., Gartner G., Karimi H., Rizos C. (2007). Applications of location–based services: A selected review. J. Locat. Based Serv..

[2] Azuma R., Baillot Y., Behringer R., Feiner S., Julier S., MacIntyre B. (2001). Recent advances in augmented reality. IEEE Comput. Graph. Appl..

[3] Majumder S., Mondal T., Deen M.J. (2017). Wearable Sensors for Remote Health Monitoring. Sensors.

[4] Alam M.R., Reaz M.B.I., Ali M.A.M. (2012). A review of smart homes—Past, present, and future. IEEE Trans. Syst. Man Cybern. Part C Appl. Rev..

[5] Jang H.J., Shin J.M., Choi L. Geomagnetic Field Based Indoor Localization Using Recurrent Neural Networks. Proceedings of the GLOBECOM 2017—2017 IEEE Global Communications Conference.

[6] Bae H., Choi L. Large-Scale Indoor Positioning using Geomagnetic Field with Deep Neural Networks. Proceedings of the ICC 2019—2019 IEEE International Conference on Communications (ICC).

[7] Apple Inc. Accessing Hardware Features in iOS. Apple Developer Documentation. https://developer.apple.com/documentation.

[8] Wang Q., Ye L., Luo H., Men A., Zhao F., Huang Y. (2018). Pedestrian Dead Reckoning Based on Motion Mode Recognition Using a Smartphone. Sensors.

[9] Yang J., Feng X., Wang C. (2017). Real-Time Motion Tracking for Mobile Augmented/Virtual Reality Using Adaptive Visual-Inertial Fusion. Sensors.

[10] Zhou Y., Park J., Koltun V. (2018). Open3D: A Modern Library for 3D Data Processing. arXiv.

[11] Youssef M., Agrawala A. The Horus WLAN Location Determination System. Proceedings of the 3rd International Conference on Mobile Systems, Applications, and Services (MobiSys).

[12] Balaban E., Alonso R., Sankararaman S., Goebel K. Modeling, Detection, and Disambiguation of Sensor Faults. Proceedings of the AIAA Infotech@Aerospace 2011 Conference.

[13] STMicroelectronics (2023). LSM6DS3TR-C: iNEMO Inertial Module with Machine Learning Core.

[14] STMicroelectronics (2021). LIS3MDL: Ultra-Low-Power, High-Performance 3-Axis Magnetometer.

[15] Bosch Sensortec (2018). BMP280: Digital Pressure Sensor.

[16] ams-OSRAM AG (2022). TSL2591: High-Sensitivity, Wide-Dynamic-Range Digital Light Sensor.

[17] Dami A.R.J., Santos J.P.L., de Deus R.A.D. Long-Term Stability Analysis of MEMS IMU for Indoor Navigation Applications. Proceedings of the 2020 IEEE International Instrumentation and Measurement Technology Conference (I2MTC).

[18] Shin S.H., Park S., Kim J. (2021). Long-Term Drift Analysis of MEMS Magnetometers for Autonomous Systems. J. Sens..

[19] Chen Z., Zou H., Jiang H., Zhu Q., Chen Y.Z.G.Y., Liu K.J.R. (2023). WiFi RSSI based indoor localization and tracking: A survey. Artif. Intell. Rev..

[20] Dao D.V., Katsuki S., Lee C.H. Temperature Effects on RSSI of Wireless Sensor Network. Proceedings of the 2010 International Conference on Electronics, Information and Communication.

[21] Yang J., Tapia E. Efficient in-pocket detection with mobile phones. Proceedings of the 2013 ACM Conference on Pervasive and Ubiquitous Computing Adjunct Publication.

[22] Yang Y., Zhang T., Huang W. (2025). A dynamic K-nearest neighbor method based on strong access point credibility for indoor positioning. Front. Inf. Technol. Electron. Eng..

[23] Guo S., Niu G., Wang Z., Pun M.-O. Magnetic Field Strength Sequence based Indoor Localization Using Multi level Link node Models and k Nearest Neighbor. Proceedings of the ICC 2020—2020 IEEE International Conference on Communications (ICC).

[24] Li Y., He Z., Nielsen J., Lachapelle G. (2022). Analysis of Magnetic Field Measurements for Indoor Positioning. Sensors.

[25] McNicholas C., Mass C.F. (2018). Smartphone Pressure Collection and Bias Correction Using Machine Learning. J. Atmos. Ocean. Technol..

[26] Hochreiter S., Schmidhuber J. (1997). Long short-term memory. Neural Comput..

[27] Camp T., Boleng J., Davies V. (2002). A survey of mobility models for ad hoc network research. Wirel. Commun. Mob. Comput..

[28] Li Y., Zhuang Y., Lan H., Zhou Q., Niu X., El-Sheimy N. (2016). A hybrid WiFi/magnetic matching/PDR approach for indoor navigation with smartphone sensors. IEEE Commun. Lett..

